# Revertant Mutation Releases Confined Lethal Mutation, Opening Pandora's Box: A Novel Genetic Pathogenesis

**DOI:** 10.1371/journal.pgen.1004276

**Published:** 2014-05-01

**Authors:** Yasushi Ogawa, Takuya Takeichi, Michihiro Kono, Nobuyuki Hamajima, Toshimichi Yamamoto, Kazumitsu Sugiura, Masashi Akiyama

**Affiliations:** 1 Department of Dermatology, Nagoya University Graduate School of Medicine, Nagoya, Japan; 2 Department of Healthcare Administration, Nagoya University Graduate School of Medicine, Nagoya, Japan; 3 Department of Legal Medicine and Bioethics, Nagoya University Graduate School of Medicine, Nagoya, Japan; University of Pennsylvania, United States of America

## Abstract

When two mutations, one dominant pathogenic and the other “confining” nonsense, coexist in the same allele, theoretically, reversion of the latter may elicit a disease, like the opening of Pandora's box. However, cases of this hypothetical pathogenic mechanism have never been reported. We describe a lethal form of keratitis-ichthyosis-deafness (KID) syndrome caused by the reversion of the *GJB2* nonsense mutation p.Tyr136X that would otherwise have confined the effect of another dominant lethal mutation, p.Gly45Glu, in the same allele. The patient's mother had the identical misssense mutation which was confined by the nonsense mutation. The biological relationship between the parents and the child was confirmed by genotyping of 15 short tandem repeat loci. Haplotype analysis using 40 SNPs spanning the >39 kbp region surrounding the *GJB2* gene and an extended SNP microarray analysis spanning 83,483 SNPs throughout chromosome 13 in the family showed that an allelic recombination event involving the maternal allele carrying the mutations generated the pathogenic allele unique to the patient, although the possibility of coincidental accumulation of spontaneous point mutations cannot be completely excluded. Previous reports and our mutation screening support that p.Gly45Glu is in complete linkage disequilibrium with p.Tyr136X in the Japanese population. Estimated from statisitics in the literature, there may be approximately 11,000 p.Gly45Glu carriers in the Japanese population who have this second-site confining mutation, which acts as natural genetic protection from the lethal disease. The reversion-triggered onset of the disesase shown in this study is a previously unreported genetic pathogenesis based on Mendelian inheritance.

## Author Summary

Loss of gene functions due to nonsense mutations is a typical pathogenic mechanism of hereditary diseases. They may, however, in certain genetic contexts, confine the effects of other dominant pathogenic mutations and suppress disease manifestations. We report the first instance in the literature where the reversion of a “confining” nonsense mutation in *GJB2* gene released the dominant pathogenic effect of a coexsisting gain-of-function mutation, eliciting the lethal form of keratitis-ichthyosis-deafness syndrome (KID). We describe this form of KID syndrome caused by the reversion of the *GJB2* nonsense mutation p.Tyr136X that would otherwise have confined the effect of another dominant lethal mutation, p.Gly45Glu, in the same allele. The patient's mother had the identical misssense mutation which was confined by the nonsense mutation. An epidemiologic estimation demonstrates that approximately 11,000 individuals in the Japanese population may have the same lethal *GJB2* mutation, nonetheless protected from the manifestation of the syndrome because they also inherit the common “confining” nonsense mutation. The reversion-triggered onset of the disease shown in this study is a previously unreported genetic pathogenesis based on Mendelian inheritance.

## Introduction

A nonsense mutation may, in theory, disrupt and thus “confine” the effects of another dominant pathogenic mutation when the two mutations coexist in the same allele of a single gene. Furthermore, in such cases, reversion of the confining nonsense mutation may paradoxically elicit a congenital disease, although proven cases of this hypothetical pathogenesis have not been reported.

Keratitis-ichthyosis-deafness (KID) syndrome (OMIM 148210) is a rare congenital ectodermal disorder characterized by vascularizing keratitis, ichthyosiform erythroderma and sensorineural hearing loss [1]. KID syndrome is mainly caused by a heterozygous germ line missense mutation in GJB2 (Entrez Gene ID: 2706) encoding connexin 26 (Cx26) (RefSeq: NM_004004.5) [2]–[4].

Here we report a case of KID syndrome where the reversion of a missense mutation induced a lethal disease. We encountered a girl with KID syndrome from obviously healthy parents, and sequence analysis of *GJB2* revealed a heterozygous missense mutation, p.Gly45Glu, in the patient. Unexpectedly, her healthy mother also had the heterozygous missense mutation p.Gly45Glu, as well as another heterozygous nonsense mutation: p.Tyr136X. From these findings, we hypothesized that the p.Tyr136X mutation confines the pathogenic effect of p.Gly45Glu in the mother and that the reversion of p.Tyr136X triggered the onset of KID syndrome in the patient. In the present study, TA cloning and haplotype analysis of the family confirmed that an allelic recombination event involving the maternal allele carrying the two mutations generated the pathogenic allele unique to the patient. Furthermore, cotransfection experiments and a neurobiotin uptake assay clearly demonstrated that the p.Tyr136X mutation confines the pathogenic effects of the p.Gly45Glu mutation. Thus, to our knowledge, the present findings provide the first evidence of reversion-triggered onset of a congenital disease.

## Results

### The Patient's Mother Had Both *GJB2* Lethal Missense Mutation and Confining Nonsense Mutation, Although the Patient Had Only the Lethal One

**Figure 1:**
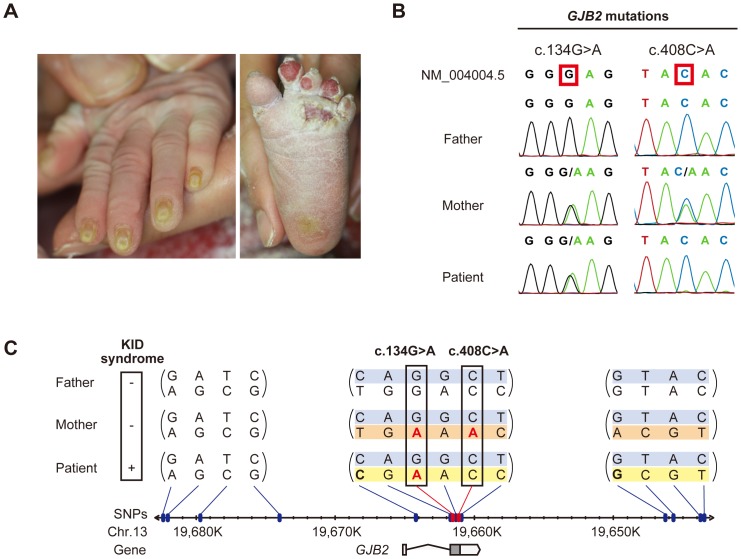
Sequence and haplotype analysis of the present case of KID syndrome. (A) Clinical manifestations of the patient are shown. Marked hyperkeratosis of the palms and soles is seen. (B) Identification of c.134G>A and c.408C>A mutations in the patient and her parents. The patient is compound heterozygous for the two mutations. (C) Haplotype analysis of the family members. Fourteen heterozygous SNPs spanning >39 kbp surrounding the *GJB2* gene are identified and assembled into three contigs (shown in parenthesis). The nucleotides altered by the c.134G>A and c.408C>A mutations are boxed. The altered nucleotides are in red. The patient and her parents share a single haplotype (top; blue background). The patient has a unique haplotype (bottom; yellow background) that is not harbored by either parent. The maternally unique haplotype is shown in orange.

The KID syndrome patient is a girl born from apparently healthy Japanese parents. She showed ichthyosiform erythroderma at birth, and later she developed typical manifestations that lead to the diagnosis of KID syndrome (Figure 1A). Despite intensive care, she died of the disease.

**Figure 2:**
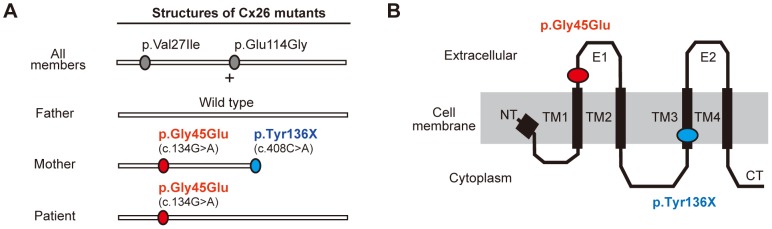
Configurations and topological mapping of the *GJB2* mutations in the family. (A) Structures of Cx26 mutants. The mutations/variants found in each allele of the family members are shown. p.Val27Ile and p.Glu114Gly are non-pathological variants. (B) Topological mapping of the Cx26 mutations. p.Gly45Glu (red) is located in the first extracellular loop domain and is thought to affect the channel activity of gap junctions. TM1–4: transmembrane domain 1–4; E1–2: extracellular domain 1–2; NT: N terminus; CT: C terminus.

Sequence analysis of *GJB2* was performed to confirm the diagnosis. Direct sequencing of PCR fragments spanning all the exons of *GJB2* revealed a heterozygous missense mutation, c.134G>A (p.Gly45Glu), in exon 2 of *GJB2* in the patient and her mother, but not in her father (Figure 1B). Her mother had an additional heterozygous nonsense mutation, c.408C>A (p.Tyr136X), in the same exon (Figure 1B). TA cloning analysis showed that the c.408C>A and c.134G>A mutations were in cis configuration. All family members uniformly harbored the two known non-pathological SNPs [5] c.79G>A (p.Val27Ile) (rs2274084) and c.341A>G (p.Glu114Gly) (rs2274083) heterozygously and in trans configuration with the c.134G>A or c.408C>A mutation (Figure 1C and 2A). The existence of the *GJB2* mRNA harboring the c.134G>A missense mutation in the patient's skin was verified by a RT-PCR assay (Figure S1). To confirm the biological relationship between the patient and her parents, we genotyped for 15 short tandem repeat (STR) loci with tetranucleotide repeat units using a multiplex kit. Since all of the genotypes for 15 STR loci were consistent with the relationship between the parents and child and each combined probability of exclusion and paternity was calculated as 0.999999997 and 0.9999999986, respectively, the authenticity of biological relationship between the parents and the child was confirmed accurately (Tables S1 and S2).

### Revertant Mutation of Confining Nonsense Mutation Occurred in the Patient's Pathogenic Allele of *GJB2*

To elucidate the origin of the c.134G>A mutation in the patient, haplotype analysis was performed. Forty SNPs annotated by the International HapMap Project [6] spanning the >39 kbp region surrounding the *GJB2* gene were sequenced. Fourteen SNPs were found to be heterozygous in one or more of the family members (Figure 1C and S2). TA cloning analysis mapped the heterozygous SNPs into three separate genetic regions (Figure 1C). All family members had at least one common haplotype in each genetic region, suggesting that they share a haplotype in the >39 kb genetic region we studied. Unexpectedly, the patient harbored a unique haplotype that was not seen in either of her parents (Figure 1C). No evidence of spontaneous mutations was found besides these SNP sites through the direct sequencing of the entire coding region of *GJB2*.

We performed an extended SNP microarray analysis spanning 83,483 SNPs throughout chromosome 13. No apparent chromosomal aberration was detected besides a 1,430 kbp copy-number neutral loss-of-heterozygosity region on 13q31.1 which was unique to the patient's genome.

From these findings, we reasoned that an allelic recombination event involving the shared allele (Figure 1C, shown in blue) and the maternally unique allele (Figure 1C, shown in orange) generated the haplotype unique to the patient (see also the Discussion section below), since it differs by three or more base pairs from the counterparts carried by either parent, giving only a remote possibility of coincidental accumulation of spontaneous point mutations at these specific SNP sites. The latter possibility, however, cannot be completely excluded.

The blood cells of the patient did not show mosaicism, and the patient's skin symptoms were fairly evenly distributed over the entire body surface. These findings suggest that the patient was not mosaic for the *GJB2* mutation. Thus, we consider the reversion leading to the pathogenic allele in the patient to be a pre-zygotic event.

### Gap Junctions Containing p.Gly45Glu-Mutant Connexin 26 (CX26) Showed Aberrant Gating Activity, Whereas p.Gly45Glu/p.Tyr136X-Mutant CX26 Were Excluded from Functional Gap Junction Formation

As described above, the patient who harbored the p.Gly45Glu mutation manifested the disease, while the mother who harbored the mutations p.Gly45Glu and p.Tyr136X was apparently unaffected (Figure 2A). A heterozygous *de novo* p.Gly45Glu mutation is known to cause the lethal form of KID syndrome [3], and its molecular pathogenic mechanism has been well described [7]–[9]. Cx26, the product of *GJB2*, is a gap junction protein with four transmembrane domains and two extracellular domains (Figure 2B). The Cx26 molecule is a protomer of a hexameric connexon, and two connexons expressed on the membranes of neighboring cells connect to form a gap junction channel [10]. Gly45 locates at a domain that lines the channel pore and probably mediates voltage sensing [10]. Connexons containing p.Gly45Glu mutants function as hemichannels with aberrantly increased activity [7], [8] that leads to the disease manifestations 3,[9]. It is also known that, besides KID syndrome, biallelic loss of function of *GJB2* causes autosomal recessive non-syndromic hearing loss (NSHL) [11]. The fact that the p.Gly45Glu/p.Tyr136X mutation homozygously or compound heterozygously causes NSHL suggests that this mutation leads to total loss of function for the *GJB2* product [5].

These considerations lead us to hypothesize that the p.Tyr136X mutation confines and rescues the dominant pathogenic effect of the p.Gly45Glu mutation. Since inter-protomer interactions of Cx26 require the fourth transmembrane domain [10] that is terminated by the p.Tyr136X mutation (Figure 2 A and B), a Cx26 carrying p.Gly45Glu/p.Tyr136X alteration would be excluded from the hexameric connexons.

This phenomenon, in which a second-site mutation cancels an exsisting pathogenic mutation, was previously reported; it is called “partial reversion”, because the wild-type allele itself is not attained, although the seoncd-site mutation rescues the disease [12].

**Figure 3:**
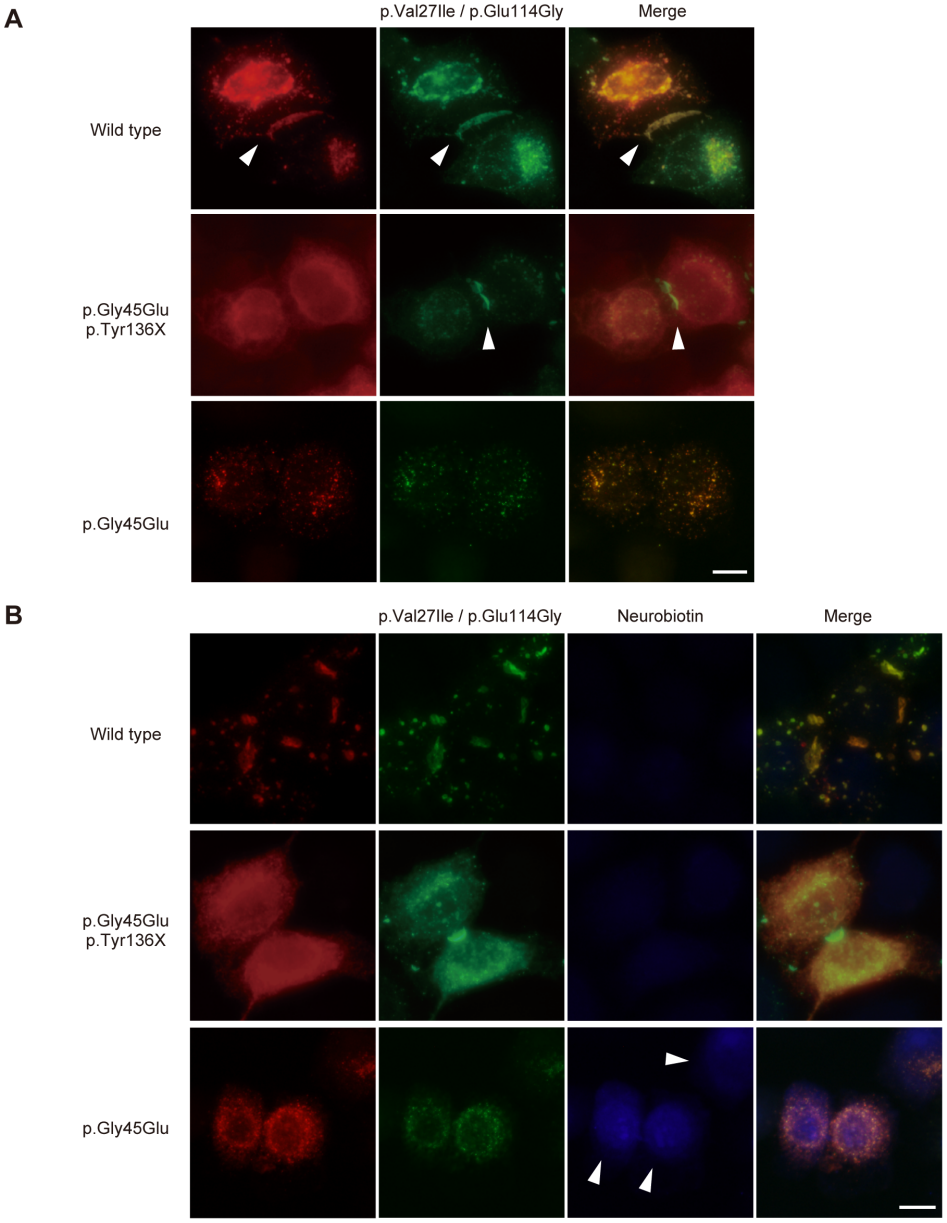
The p.Tyr136X mutation confines the effect of the pGly45Glu mutation. (A) Gap junction formation by the transfected Cx26 variants. Each panel contains two co-transfected cells connected to each other. Wild-type, p.Gly45Glu/p.Tyr136X and p.Gly45Glu mutants of Cx26 were tagged with monomeric Red Fluorescent Protein (mRFP) and co-transfected with Green Fluorescent Protein (EGFP)-tagged Cx26 p.Val27Ile/p.Glu114Gly into HeLa cells as indicated. Gap junction formation sites are indicated by arrowheads. The combination of WT Cx26 and Cx26 p.Val27Ile/p.Glu114Gly (top row) results in gap junctions that consist of both Cx26 proteins (yellow signal). The combination of Cx26 p.Gly45Glu/p.Tyr136X and Cx26 p.Val27Ile/p.Glu114Gly (middle row) results in gap junctions with only Cx26 p.Val27Ile/p.Glu114Gly (green signal). No apparent gap junction formation is seen when Cx26 p.Gly45Glu and Cx26 p.Val27Ile/p.Glu114Gly are cotransfected (bottom row). (B) Aberrant gate opening detected with neurobiotin uptake assay. Fluorescent-tagged Cx26s were cotransfected into HeLa cells as indicated, and treated with neurobiotin in a calcium-free condition. Uptake was detected with AlexaFluor350 streptoavidin dye (blue). Aberrant uptake of neurobiotin is observed only in cells cotransfected with Cx26 p.Gly45Glu and Cx26 p.Val27Ile/p.Glu114Gly (bottom row).

To test this hypothesis, we observed the colocalization of fluorescent-tagged Cx26 variants in HeLa cells. The father had wild-type and p.Val27Ile/p.Glu114Gly variant alleles (Figure 2A). When these Cx26s were cotransfected, they together formed gap junctions, suggesting that both proteins retain their native functions (Figure 3A). The Cx26 p.Gly45Glu/p.Tyr136X mutant failed to enter the gap junction generated by Cx26 p.Val27Ile/p.Glu114Gly, demonstrating that only the latter form comprises the functional gap junctions in the mother (Figure 3A). Cx26 p.Gly45Glu colocalized with the p.Val27Ile/p.Glu114Gly variant but failed to form gap junctions (Figure 3A). In a neurobiotin uptake assay, which monitors channel activity as cellular uptake of a neurobiotin tracer [9], only the p.Gly45Glu mutant and not the p.Gly45Glu/p.Tyr136X mutant induced the aberrant uptake (Figure 3B).

## Discussion

Many cases of revertant mosaicism have been reported as “natural gene therapy” where the mitotic recombination results in revertant mutations that mitigate the disease symptoms [13]–[16]. However, the present study is the first report to demonstrate a mutant reversion triggering a genetic disease.

The present data of genomic DNA sequencing and haplotype analysis demonstrate that the patient and her father share an identical haplotype (Figure 1C, shown in blue). We hypothesized that the entire blue allele in the patient's genome was derived from the father, while the other allele (Figure 1C, shown in yellow) was basically derived from the mother. It seemed, however, that this allele underwent pre-zygotic reversion during meiosis of the maternal gamete. The fact that the patient's unique allele (Figure 1C, shown in yellow) differs by three non-continuous SNPs from the unique maternal allele (Figure 1C, shown in orange) while the neighboring SNPs are conserved might be explained by multiple events of gene conversion involving both of the maternal alleles (Figure 1C, shown in blue and orange) that may have occurred in this genetic region.

Double cross-over also might account for the recombination, but it is less likely, considering that the non-conserved and conserved SNPs in the patient's allele reside within close proximity (Figure 1C); the average length of the gene conversion tract is estimated to be in the range of 55–290 bp, whereas the cross-over tracts are typically longer [17]. Mitotic gene conversion has been found in some cases of revertant mosaicism in cutaneous disease, including generalized atrophic benign epidermolysis bullosa [12], [13]. We are unaware of any previous report of multiple gene conversions within a relatively short genetic segment as in the present case. However, the present data compel us to consider that it occurred. Since the patient's unique allele differs by three or more base pairs from the counterparts carried by either parent, we judge the possibility of coincidental accumulation of spontaneous point mutations at these specific SNP sites to be highly unlikely. This possibility, however, cannot be completely excluded.

**Figure 4:**
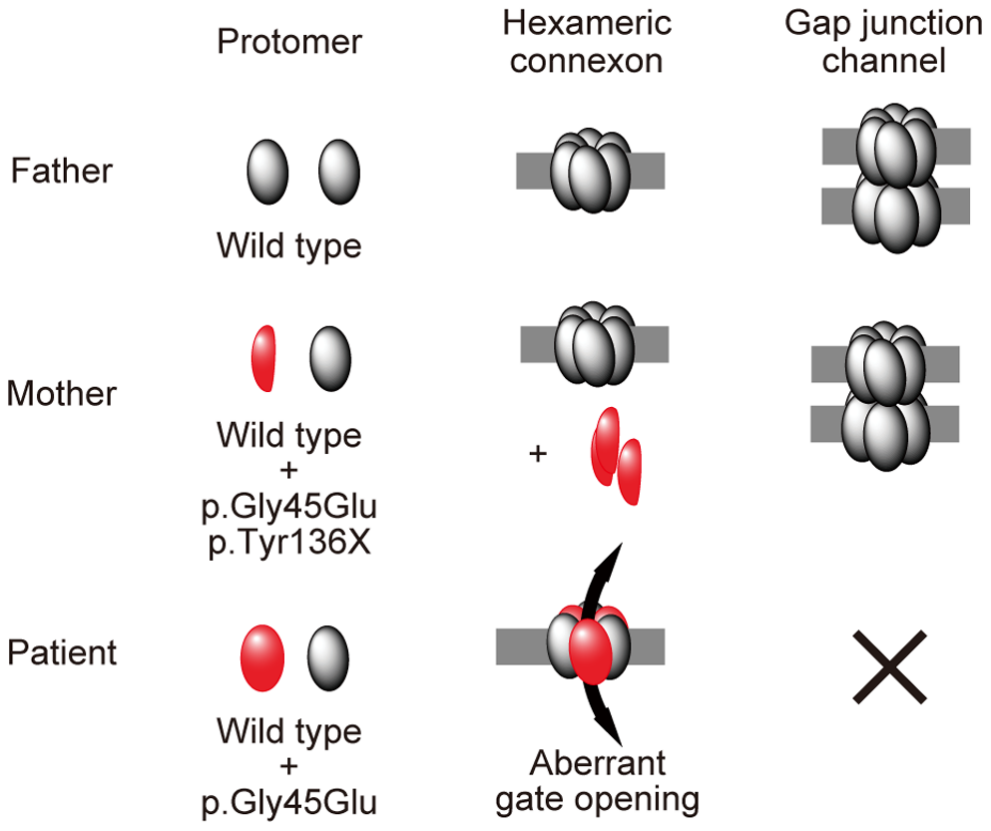
Schematic of the mechanism whereby the p.Tyr136X mutation confines the effect of the p.Gly45Glu mutation. The truncated Cx26 peptides produced from the mutant p.Gly45Glu/p.Tyr136X are not incorporated into connexons or gap junctions (middle row), although Cx26 peptides derived from the mutant p.Gly45Glu are incorporated into connexons, resulting in aberrant gate opening and malformation of gap junctions (bottom row).

As evidence supporting our hypothesis, consistent with a previous report [9], we clearly demonstrated that Cx26 p.Gly45Glu colocalized with the p.Val27Ile/p.Glu114Gly variant but failed to form gap junctions (Figure 3A). Previous studies have shown that Cx26 p.Gly45Glu forms hemichannels that are aberrantly activated at low extracellular Ca2+ levels [9]. The present study used a neurobiotin uptake assay [9] to show that only the p.Gly45Glu mutant and not the p.Gly45Glu/p.Tyr136X mutant induces the aberrant uptake (Figure 3B). These results taken together support the model in which the p.Tyr136X mutation confines the dominant gain-of-function effect of the p.Gly45Glu mutation to prevent the onset of the disease (Figure 4). Such secondary effects of revertants may pose a challenge in genetic analyses of extended genes or more than one gene with functional interactions.

Interestingly, in the group of Japanese patients with bilateral sensorineural hearing loss, it is not uncommon to find *GJB2* p.Gly45Glu carriers, but none of them are affected by KID syndrome [5]. They uniformly have a tandem p.Tyr136X mutation, as in the mother of the present case [5]. Thus, we hypothesized that, in the Japanese population, carriers of p.Gly45Glu are protected from the lethal form of KID syndrome by the tandem, confining mutation p.Tyr136X.

To clarify the frequency of the p.Gly45Glu mutation in the entire Japanese population, we performed screening analysis for the two mutations p.Gly45Glu and p.Tyr136X in a normal control group consisting of 920 overall healthy Japanese individuals (1,840 alleles). Neither p.Gly45Glu nor p.Tyr136X was found in any of the 1,840 alleles (data not shown). Tsukada *et al.* [5] also reported that neither p.Gly45Glu nor p.Tyr136X was found in 252 Japanese healthy control individuals (504 control Japanese alleles). These results indicate that the alleles with tandem p.Gly45Glu and p.Tyr136X mutations are infrequent in the general Japanese population. However, in the epidemiological statistics of Tsukada *et al.* [5], we found screening data for *GJB2* mutations in Japanese patients with sensorineural hearing loss. The report revealed that, among 1,343 Japanese patients with hearing loss, 33 patients had one or two p.Gly45Glu alleles (34 p.Gly45Glu alleles in 2686 alleles for an allele frequency of 1.27%; 33 carriers in 1,343 patients for a carrier rate of 2.46%). This means 2.46% of Japanese patients with bilateral sensorineural hearing loss have one or two p.Gly45Glu alleles. As for the prevalence of sensorineural hearing loss, it was reported that 3.5 per 1,000 individuals in the entire population have bilateral sensorineural hearing loss [18]. Thus, calculating from these epidemiological statistics, we estimate that 8.6 per 100,000 individuals, or approximately 11,000 individuals in the entire Japanese population, have one or two p.Gly45Glu alleles. However, no patient with the lethal form of KID syndrome due to p.Gly45Glu has been reported in the Japanese population as far as we know, although the mutation p.Gly45Glu has been reported as a cause of the lethal form of KID syndrome in several European patients [3], [19]–[22].

Tsukada *et al.* [5] reported that all 34 alleles with p.Gly45Glu found in the Japanese patients with sensorineural hearing loss also had p.Tyr136X, suggesting that p.Gly45Glu is in complete linkage disequillibrium with p.Tyr136X in the Japanese population. In our mutation screening, no allele carrying either or both mutations, p.Gly45Glu and p.Tyr136X, was found in 920 Japanese individuals (1,840 alleles) and these results support the idea that p.Gly45Glu is in complete LD with p.Tyr136X in the Japanese population.

In light of this, we conclude that, even though individuals may have the dominant lethal mutation p.Gly45Glu, the confining mutation p.Tyr136X *in cis* configuration protects against the disease, KID syndrome, in the approximately 11,000 Japanese individuals in the entire Japanese population who harbor p.Gly45Glu. The allele with the tandem mutations p.Gly45Glu and p.Tyr136X causes hearing loss in an autosomal recessive manner. Most carriers of the tandem mutations in the Japanese population are heterozygous for the allele, such as the patient's mother in the present study, and are not affected with hearling loss.

In summary, our findings demonstrate that the second-site confining mutation is an imporatant genetic protection mechanism, and its loss, like the opening of Pandora's box, is a novel genetic pathogenesis that releases the hidden genetic disease.

## Materials and Methods

### Ethics Statement

This study was approved by the Bioethics Committee of the Nagoya University Graduate School of Medicine and was conducted according to The Declaration of Helsinki Principles. Written informed consent was obtained from the parents.

### The Patient and Her Parents

The patient was referred and seen at the Outpatient Clinic of Dermatology, Nagoya University Hospital.

### Sequence Analysis and TA Cloning

Genomic DNA extracted from peripheral blood was used as a template for PCR amplification, followed by direct automated sequencing. The entire coding regions of *GJB2* including the exon/intron boundaries were sequenced as reported elsewhere [4]. TOPO-TA cloning kit (Life Technologies) was used for TA cloning analyses. PCR primers were designed to amplify the genetic regions containing the selected SNPs, and the acquired PCR products were analyzed by direct sequencing. For amplification of PCR fragments longer than 1,000 base pairs, KOD-Plus-Neo polymerase (Toyobo) or PrimeScript GXL polymerase (Takara Bio) was used and the PCR products were cloned with the TOPO XL-TA Cloning Kit (Life Technologies) after the addition of a 3′-adenine overhang.

### Verification of the Parent-Child Relationship

The parent-child relationship was validated using AmpFlSTR Identifier plus PCR amplification kit (Applied Biosystems) according to the manufacturer's instructions. The combined probability of exclusion and the combined probability of paternity (in the case of the odds ratio for prior probability = 1) for 15 STR loci were calculated to confirm the authenticity of the biological relationship between the parents and the child.

### Genotyping of *GJB2* Mutations in 920 Japanese Control Individuals

Genomic DNA was extracted from whole blood using the QIAamp DNA Blood Maxi Kit (Qiagen). Real-time PCR-based genotyping of the *GJB2* mutations was performed with TaqMan MGB probe genotyping assay according to the manufacturer's instructions provided by Roche Diagnostics. To detect an allele of each mutation, a set of two TaqMan MGB probes labeled with a fluorescent dye (FAM or VIC) and a quencher dye (non-fluorescent dye; NFD) followed by minor groove binder (MGB), and sequence-specific forward and reverse primers were synthesized by Life Technologies Corporation. The sequences of assay probes/primers are shown in Table S3 in this article's supplementary material. Real-time PCR was performed with LightCycler 480 system II 384 plate (Roche Diagnostics) in a final volume of 5 µl containing 2× LightCycler 480 Probes Master (Roche Diagnostics), 200 nM probes for wild type and mutant each and 900 nM forward and reverse primers each, with 5 ng genomic DNA as the template. The thermal conditions were the following: 95°C for 10 min, followed by 45 cycles of 95°C for 10 s, 60°C for 60 s and 72°C for 1 s, with a final cooling at 40°C for 30 s. Endpoint fluorescence was measured for each sample well. Afterward, genotyping was performed using endpoint genotyping analysis in LightCycler 480 software.

Eight hundred and twenty controls were analyzed with the real-time PCR-based genotyping of *GJB2* mutations, and another 100 controls were analyzed with the direct automated sequencing for the entire coding region of *GJB2*.

### *GJB2* Expression Study

Total RNA from the formaldehyde-fixed paraffin-embedded skin sample of the patient was extracted using the RNeasy FFPE kit (Qiagen) and Deparaffinization Solution (Qiagen) according to the manufacturer's instructions. The total RNA was reverse-transcribed with a *GJB2* specific primer, 5′-GGATGTGGGAGATGGGGAAGTAGTG-3′, using PrimeScriptII 1st strand cDNA synthesis kit (Takara, Japan). The PCR fragment harboring c.134G>A mutation was amplified with primer sets, 5′-GGAAAGATCTGGCTCACCGTCCTC-3′ and 5′-CGTAGCACACGTTCTTGCAGCCTG-3′, and directly sequenced with the same primers.

### SNP Array

Chromosome-wide genotyping was performed using HumanOmni2.5–8 BeadChip (Illumina), which covers a total of 2,379,855 SNPs throughout the genome, including 83,482 SNPs on chromosome 13. Genomic DNA was hybridized according to the manufacturer's instructions and data analysis was carried out using GenomeStudio software (Illumina).

### Cell Culture

HeLa cells were cultured in Dulbecco's modified Eagle's medium (DMEM) containing 10% fetal calf serum. For transfection of plasmids, cells were seeded onto 8-well LabTek chamber slides (Thermo Scientific) and transfected with FuGene HD Transfection Reagent (Roche Applied Science) according to the manufacturer's instructions.

### Plasmid Construction

The coding sequences of Cx26 variants were amplified from the genome of the patient or the parents, fused to cDNAs coding enhanced green fluorescent protein (EGFP) (Clontech) or monomeric red fluorescent protein (Clontech), and subcloned into pcDNA3.1(-) plasmid using the InFusion HD Cloning Kit (Takara Bio). The coding sequences of the generated vectors were checked for PCR errors by direct sequencing.

### Colocalization Assay

HeLa cells were cotransfected with the EGFP-tagged and mRFP-tagged vectors. Forty-eight hours after transfection, the cells were fixed with 4% formaldehyde. Fluorescent images were obtained using FSX-100 microscope system (Olympus).

### Neurobiotin Uptake Assay

HeLa cells were cotransfected with EGFP-tagged and mRFP-tagged Cx26 variant expressing vectors, and neurobiotin uptake assay was performed as described elsewhere [9]. Briefly, cells were washed with calcium free Hank's buffered salt solution for 20 minutes and incubated with phosphate-buffered saline (PBS) containing 0.1 mg/ml neurobiotin (Vector Laboratories) for another 20 minutes. Cells were washed three times with PBS supplemented with 2 mM CaCl_2_ for 10 minutes at 37°C. The cells were fixed with 4% formaldehyde and permeabilized and blocked with 3% BSA/0.1% Triton X-100/PBS for 1 hour. Then the cells are stained with 3% BSA/0.1% Triton X-100/PBS containing 10 µg/ml Alexa Fluor 350-streptoavidin (Life Technologies) for 1 hour, followed by three washes with 0.1% Triton X-100/PBS. Stained cells were fixed with ProLong Gold antifade reagent (Life Technologies) and fluorescent images were obtained.

## Supporting Information

Figure S1 The *GJB2* mRNA harboring the missense mutation is expressed in the patient's skin. (A) RT-PCR from the total RNA extracted from a formaldehyde-fixed paraffin-embedded skin sample of the patient. A 136-bp PCR fragment was amplified from the *GJB2* cDNA obtained from the skin sample of the patient. (B) Detection of *GJB2* cDNA harboring the c.134G>A missense mutation. The PCR fragment was directly sequenced to confirm the expression of the mutant *GJB2* mRNA.
(TIF)

Figure S2 Summary of 40 SNPs spanning the >39 kbp region including GJB2. The SNPs inside the red box reside within the GJB2 gene. Note that the nucleotides are in the strand opposite those shown in Figure 1.
(TIF)

Table S1 Genotyping of the patient and her parents for 15 short tandem repeat (STR) loci.
(DOCX)

Table S2 The combined probability of exclusion and the combined probability of paternity.
(DOCX)

Table S3 The sequence of probes/primers for real-time PCR-based genotyping of *GJB2* mutations.
(DOCX)

## References

[1] Skinner BA, Greist MC, Norins AL (1981) The keratitis, ichthyosis, and deafness (KID) syndrome. Arch Dermatol 117: 285–289. 7224657

[2] Richard G, Rouan F, Willoughby CE, Brown N, Chung P, et al (2002) Missense mutations in GJB2 encoding connexin-26 cause the ectodermal dysplasia keratitis-ichthyosis-deafness syndrome. Am J Hum Genet 70: 1341–1348. 11912510 10.1086/339986 PMC447609

[3] Janecke AR, Hennies HC, Gunther B, Gansl G, Smolle J, et al (2005) GJB2 mutations in keratitis-ichthyosis-deafness syndrome including its fatal form. Am J Med Genet A 133A: 128–131. 15633193 10.1002/ajmg.a.30515

[4] Arita K, Akiyama M, Aizawa T, Umetsu Y, Segawa I, et al (2006) A novel N14Y mutation in Connexin26 in keratitis-ichthyosis-deafness syndrome: analyses of altered gap junctional communication and molecular structure of N terminus of mutated Connexin26. Am J Pathol 169: 416–423. 16877344 10.2353/ajpath.2006.051242 PMC1698798

[5] Tsukada K, Nishio S, Usami S (2010) A large cohort study of GJB2 mutations in Japanese hearing loss patients. Clin Genet 78: 464–470. 20497192 10.1111/j.1399-0004.2010.01407.x

[6] The International HapMap Consortium (2003) The International HapMap Project. Nature 426: 789–796. 14685227 10.1038/nature02168

[7] Stong BC, Chang Q, Ahmad S, Lin X (2006) A novel mechanism for connexin 26 mutation linked deafness: cell death caused by leaky gap junction hemichannels. Laryngoscope 116: 2205–2210. 17146396 10.1097/01.mlg.0000241944.77192.d2

[8] Gerido DA, DeRosa AM, Richard G, White TW (2007) Aberrant hemichannel properties of Cx26 mutations causing skin disease and deafness. Am J Physiol Cell Physiol 293: C337–345. 17428836 10.1152/ajpcell.00626.2006

[9] Mese G, Sellitto C, Li L, Wang HZ, Valiunas V, et al (2011) The Cx26-G45E mutation displays increased hemichannel activity in a mouse model of the lethal form of keratitis-ichthyosis-deafness syndrome. Mol Biol Cell 22: 4776–4786. 22031297 10.1091/mbc.E11-09-0778 PMC3237621

[10] Maeda S, Nakagawa S, Suga M, Yamashita E, Oshima A, et al (2009) Structure of the connexin 26 gap junction channel at 3.5 A resolution. Nature 458: 597–602. 19340074 10.1038/nature07869

[11] Kalatzis V, Petit C (1998) The fundamental and medical impacts of recent progress in research on hereditary hearing loss. Hum Mol Genet 7: 1589–1597. 9735380 10.1093/hmg/7.10.1589

[12] Jonkman MF (1999) Revertant mosaicism in human genetic disorders. Am J Med Genet 85: 361–364. 10398261 10.1002/(sici)1096-8628(19990806)85:4<361::aid-ajmg11>3.0.co;2-e

[13] Jonkman MF, Scheffer H, Stulp R, Pas HH, Nijenhuis M, et al (1997) Revertant mosaicism in epidermolysis bullosa caused by mitotic gene conversion. Cell 88: 543–551. 9038345 10.1016/s0092-8674(00)81894-2

[14] Jonkman MF, Castellanos Nuijts M, van Essen AJ (2003) Natural repair mechanisms in correcting pathogenic mutations in inherited skin disorders. Clin Exp Dermatol 28: 625–631. 14616831 10.1046/j.1365-2230.2003.01400.x

[15] Choate KA, Lu Y, Zhou J, Choi M, Elias PM, et al (2010) Mitotic recombination in patients with ichthyosis causes reversion of dominant mutations in KRT10. Science 330: 94–97. 20798280 10.1126/science.1192280 PMC3085938

[16] Kiritsi D, He Y, Pasmooij AM, Onder M, Happle R, et al (2012) Revertant mosaicism in a human skin fragility disorder results from slipped mispairing and mitotic recombination. J Clin Invest 122: 1742–1746. 22466645 10.1172/JCI61976 PMC3336993

[17] Jeffreys AJ, May CA (2004) Intense and highly localized gene conversion activity in human meiotic crossover hot spots. Nat Genet 36: 151–156. 14704667 10.1038/ng1287

[18] Morton CC, Nance WE (2006) Newborn hearing screening–a silent revolution. N Engl J Med 354: 2151–2164. 16707752 10.1056/NEJMra050700

[19] Griffith AJ, Yang Y, Pryor SP, Park HJ, Jabs EW, et al (2006) Cochleosaccular dysplasia associated with a connexin 26 mutation in keratitis-ichthyosis-deafness syndrome. Laryngoscope 116: 1404–1408. 16885744 10.1097/01.mlg.0000224549.75161.ca PMC2563154

[20] Jonard L, Feldmann D, Parsy C, Freitag S, Sinico M, et al (2008) A familial case of Keratitis-Ichthyosis-Deafness (KID) syndrome with the GJB2 mutation G45E. Eur J Med Genet 51: 35–43. 18024254 10.1016/j.ejmg.2007.09.005

[21] Sbidian E, Feldmann D, Bengoa J, Fraitag S, Abadie V, et al (2010) Germline mosaicism in keratitis-ichthyosis-deafness syndrome: pre-natal diagnosis in a familial lethal form. Clin Genet 77: 587–592. 20412116 10.1111/j.1399-0004.2009.01339.x

[22] Koppelhus U, Tranebjaerg L, Esberg G, Ramsing M, Lodahl M, et al (2011) A novel mutation in the connexin 26 gene (GJB2) in a child with clinical and histological features of keratitis-ichthyosis-deafness (KID) syndrome. Clin Exp Dermatol 36: 142–148. 20846357 10.1111/j.1365-2230.2010.03936.x

