# Supplementary figures and images for: Revertant Mutation Releases Confined Lethal Mutation, Opening Pandora's Box: A Novel Genetic Pathogenesis
Source: PLoS Genet. 2014 May 1;10(5):e1004276. doi: 10.1371/journal.pgen.1004276 (PMC4006701; doi:10.1371/journal.pgen.1004276)

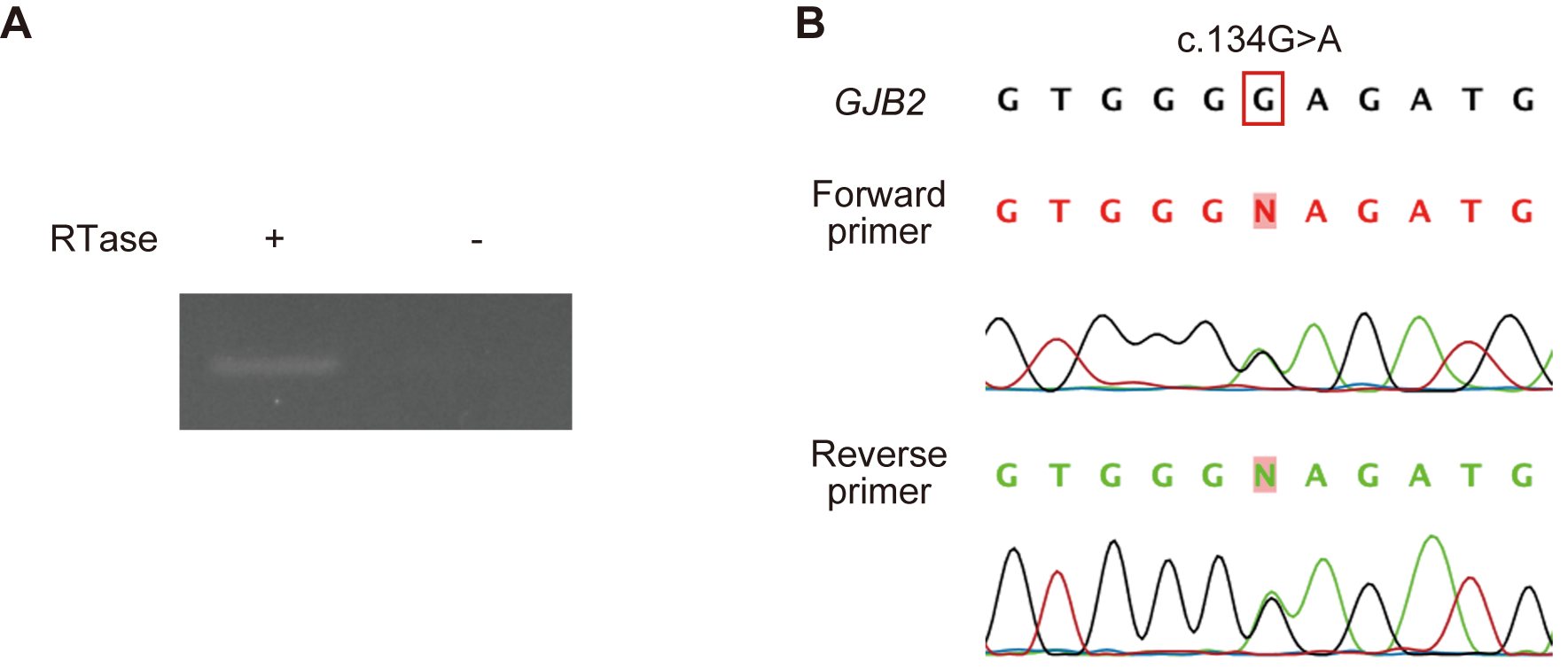

Supplement: Figure S1 — The GJB2 mRNA harboring the missense mutation is expressed in the patient's skin. (A) RT-PCR from the total RNA extracted from a formaldehyde-fixed paraffin-embedded skin sample of the patient. A 136-bp PCR fragment was amplified from the GJB2 cDNA obtained from the skin sample of the patient. (B) Detection of GJB2 cDNA harboring the c.134G>A missense mutation. The PCR fragment was directly sequenced to confirm the expression of the mutant GJB2 mRNA. (TIF) [file pgen.1004276.s001.tif]

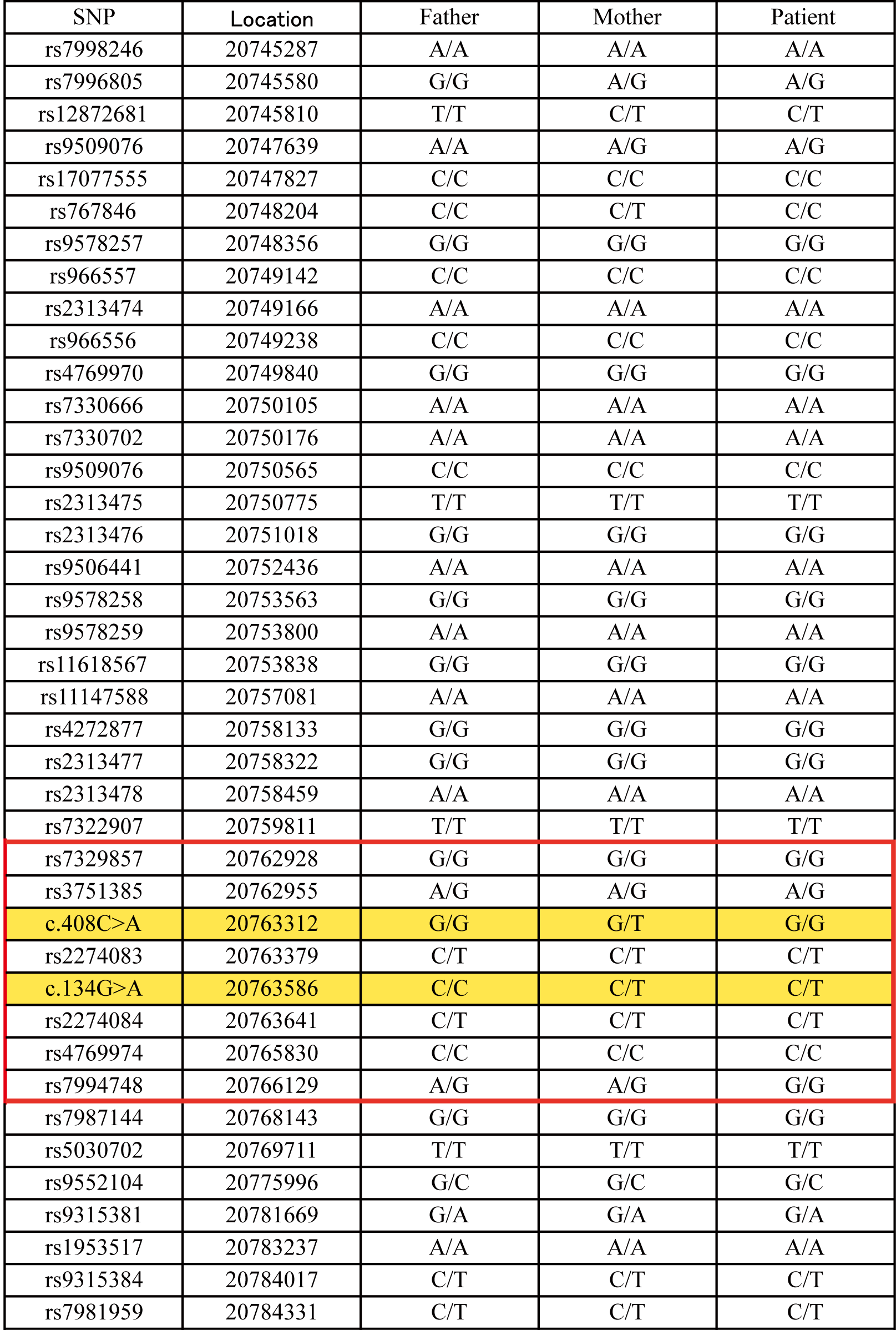

Supplement: Figure S2 — Summary of 40 SNPs spanning the >39 kbp region including GJB2. The SNPs inside the red box reside within the GJB2 gene. Note that the nucleotides are in the strand opposite those shown in Figure 1. (TIF) [file pgen.1004276.s002.tif]
