# Supplementary material for: Revertant Mutation Releases Confined Lethal Mutation, Opening Pandora's Box: A Novel Genetic Pathogenesis
Source: PLoS Genet. 2014 May 1;10(5):e1004276. doi: 10.1371/journal.pgen.1004276 (PMC4006701; doi:10.1371/journal.pgen.1004276)
Supplement: Table S1 — Genotyping of the patient and her parents for 15 short tandem repeat (STR) loci. (DOCX) [file pgen.1004276.s003.docx]

**Table S1.** Genotyping of the patient and her parents for 15 short tandem repeat (STR) loci with tetranucleotide repeat units.

| Locus | STR type | | |
| --- | --- | --- | --- |
|  | Mother | Patient | Father |
|  |  |  |  |
|  |  |  |  |
| D8S1179 | 11,15 | 10,11 | 10,10 |
| D21S11 | 30,32.2 | 30,30.3 | 29,30.3 |
| D7S820 | 10,12 | 10,10 | 10,10 |
| CFS1PO | 10,12 | 10,12 | 10,11 |
| D3S1358 | 15,16 | 15,18 | 15,18 |
| TH01 | 9,9 | 6,9 | 6,9 |
| D13S317 | 8,10 | 8,10 | 10,12 |
| D16S539 | 10,12 | 10,10 | 9,10 |
| D2S1338 | 17,23 | 22,23 | 17,22 |
| D19S433 | 13,15 | 13,14.2 | 14.2,15 |
| vWA | 14,17 | 14,19 | 15,19 |
| TPOX | 11,11 | 9,11 | 8,9 |
| D18S51 | 14,15 | 14,15 | 14,14 |
| Amerogenin | X,X | X,X | X,Y |
| D5S818 | 11,12 | 11,12 | 11,12 |
| FGA | 23,23 | 22,23 | 18,22 |
|  |  |  |  |
|  |  |  |  |
