# Supplementary material for: Revertant Mutation Releases Confined Lethal Mutation, Opening Pandora's Box: A Novel Genetic Pathogenesis
Source: PLoS Genet. 2014 May 1;10(5):e1004276. doi: 10.1371/journal.pgen.1004276 (PMC4006701; doi:10.1371/journal.pgen.1004276)
Supplement: Table S2 — The combined probability of exclusion and the combined probability of paternity. (DOCX) [file pgen.1004276.s004.docx]

**Table S2.** The combined probability of exclusion and the combined probability of paternity.

| Locus |  | Probability of exclusion | |  | Probability of paternity | | | | |
| --- | --- | --- | --- | --- | --- | --- | --- | --- | --- |
|  |  | PE | accum. PE |  | PI | accum. PI |  | W | accum.W |
|  |  |  |  |  |  |  |  |  |  |
| D8S1179 |  | 0.7730 | 0.7730402110 |  | 8.2800 | 8.3 |  | 0.8922 | 0.8922413793 |
| D21S11 |  | 0.9856 | 0.9967226453 |  | 69.0000 | 571.3 |  | 0.9857 | 0.9982527257 |
| D7S820 |  | 0.6125 | 0.9987299476 |  | 4.6000 | 2628.1 |  | 0.8214 | 0.9996196377 |
| CFS1PO |  | 0.1366 | 0.9989034094 |  | 0.7931 | 2084.3 |  | 0.4423 | 0.9995204603 |
| D3S1358 |  | 0.8336 | 0.9998175804 |  | 5.7500 | 11984.9 |  | 0.8519 | 0.9999165687 |
| TH01 |  | 0.5974 | 0.9999265664 |  | 2.2021 | 26392.3 |  | 0.6877 | 0.9999621116 |
| D13S317 |  | 0.3914 | 0.9999553069 |  | 1.0049 | 26520.4 |  | 0.5012 | 0.9999622946 |
| D16S539 |  | 0.6745 | 0.9999854507 |  | 2.7973 | 74185.5 |  | 0.7367 | 0.9999865205 |
| D2S1338 |  | 0.8696 | 0.9999981021 |  | 7.4074 | 549522.4 |  | 0.8811 | 0.9999981802 |
| D19S433 |  | 0.8010 | 0.9999996224 |  | 4.7619 | 2616773.4 |  | 0.8264 | 0.9999996179 |
| vWA |  | 0.8425 | 0.9999999405 |  | 6.0882 | 15931532.4 |  | 0.8589 | 0.9999999372 |
| TPOX |  | 0.7561 | 0.9999999855 |  | 3.8333 | 61070874.0 |  | 0.7931 | 0.9999999836 |
| D18S51 |  | 0.4036 | 0.9999999913 |  | 2.7417 | 167439350.0 |  | 0.7327 | 0.9999999940 |
| D5S818 |  | 0.2440 | 0.9999999935 |  | 1.9761 | 330874867.3 |  | 0.6640 | 0.9999999970 |
| FGA |  | 0.5937 | 0.9999999973 |  | 2.1789 | 720958921.4 |  | 0.6854 | 0.9999999986 |
|  |  |  |  |  |  |  |  |  |  |
| Combined probability |  | 0.999999997 | |  | Combined probability | |  | 0.9999999986 | |
|  |  |  |  |  | Odds ratio for prior probability = 1 | |  |  |  |
