# Supplementary material for: Revertant Mutation Releases Confined Lethal Mutation, Opening Pandora's Box: A Novel Genetic Pathogenesis
Source: PLoS Genet. 2014 May 1;10(5):e1004276. doi: 10.1371/journal.pgen.1004276 (PMC4006701; doi:10.1371/journal.pgen.1004276)
Supplement: Table S3 — The sequence of probes/primers for real-time PCR-based genotyping of GJB2 mutations. (DOCX) [file pgen.1004276.s005.docx]

**Table S3.** The sequence of probes/primers for real-time PCR-based genotyping of *GJB2* mutations

| Mutation | Primer/Probe | Sequence |
| --- | --- | --- |
| c.134G>A | GJB2-MGB134.f | AGGGTGTTGCAGACAAAGTCG |
|  | GJB2-MGB134.r | GCATTGGAAAGATCTGGCTCA |
|  | GJB2-MGB134.probe(c) | VIC-TGCTCATCTcCCCACACC-NFD-MGB |
|  | GJB2-MGB134.probe(t) | FAM-TGCTCATCTtCCCACACC-NFD-MGB |
| c.408C>A | GJB2-MGB408.f | CGAAGATGACCCGGAAGAAG |
|  | GJB2-MGB408.r | CCAGAAGGTCCGCATCGA |
|  | GJB2-MGB408.probe(g) | VIC-TGCTGCTTGTgTAGGT-NFD-MGB |
|  | GJB2-MGB408.probe(t) | FAM-TGCTGCTTGTtTAGGT-NFD-MGB |
